# Surveillance and Epidemiology in Natural Disasters: A Novel Framework and Assessment of Reliability

**DOI:** 10.1371/currents.dis.6773eb9d5e64b733ab490f78de346003

**Published:** 2014-02-10

**Authors:** Yasmin Khan, Brian Schwartz, Ian Johnson

**Affiliations:** Public Health Ontario, Toronto, Ontario, Canada; Department of Medicine, University of Toronto, Toronto, Ontario, Canada; Public Health Ontario, Toronto, Ontario, Canada; Dalla Lana School of Public Health, Department of Family and Community Medicine, University of Toronto, Toronto, Ontario, Canada; Public Health Ontario, Toronto, Ontario, Canada; Dalla Lana School of Public Health, University of Toronto, Toronto, Ontario, Canada

## Abstract

Objective: To create a framework and methodology for organizing relevant disaster epidemiology literature. The target audience for the framework is local public health practitioners conducting emergency surveillance in the setting of preparedness or response to natural disasters. Methods: The approach to developing the framework involved utilizing the public health and emergency management literature. It was created along four axes. The first was the type of natural disaster; second was according to phase of disaster cycle; third was the impact of the disaster (health, infrastructure, economic); and fourth was related to the main outcome of the study (ie. injuries or infectious diseases). A literature review was conducted and subsequently the current literature was utilized to perform a reliability test of the established framework, using two independent reviewers. Results: Using existing disaster classification systems and risk analysis tools, a framework was developed along the four axes. The final literature search resulted in 85 articles on surveillance in natural disaster settings. The majority of studies are on the subject of hurricanes with a catastrophic impact rating. The phase of testing reliability of the framework resulted in percent agreement of 74%. Conclusions: A reliable framework was developed that enables local public health practitioners to easily access appropriate and previously utilized surveillance methods for a natural disaster emergency. This framework contributes to an evidence-informed approach to surveillance in natural disasters with public health impacts.

## Introduction

With climate change and the potential for an increasing frequency of natural disasters around the world, it is expected that there will be increasing impact on the health of populations.^1^^,^ 2 In Canada, potential hazards range from meteorological events such as hurricanes and storms to geophysical disasters like earthquakes.^3^^,^ 4 A disaster is defined as: “a sudden accident or a natural catastrophe that causes great damage or loss of life”.^5^ It is crucial that public health organizations prepare for the health consequences of potential natural disasters; furthermore, surveillance is an important component of all emergency plans. Surveillance is defined as the systematic ongoing collection, collation, and analysis of data and the timely dissemination of information to those who need to know so that action can be taken.^6^ Surveillance in the setting of natural disasters can help to identify the resulting health-related needs which in turn, will lead to the more rational and effective deployment of resources to affected populations.

One challenge is predicting in advance the type and extent of surveillance required to respond to the disaster. Changes to a jurisdiction’s surveillance system in the midst of an emergency can lead to potential biases and misinformation. In addition, inadequate surveillance due to lack of planning could inaccurately estimate the need for resources to address the issues, potentially resulting in preventable morbidity and mortality.^7^ Thus, matching the type and size of the surveillance response to the type and size of the disaster is critical to effective response delivery.

Frameworks have been increasingly used in the health emergency management field as tools to guide practice. Some frameworks pertain to medical and clinically-oriented aspects of health emergency response after the initial disaster impact such as mass casualty management and triage.^8^^,^ 9 ^,^ 10 Public health consequences of disasters such as communicable diseases and food and water insecurity generally occur after the initial disaster impact; tools have been developed to assess the public health impact of large-scale disasters.^11^^,^ 12 Some tools are focused on settings of complex humanitarian emergencies where there is a breakdown of authority as a result of conflict. This makes these frameworks less generalizable to settings that are not in conflict and have intact authority structures, but yet may still be at risk for significant impact from natural disasters.^7^^,^ 13 The World Health Organization (WHO) and other international experts have developed approaches to planning and preparedness for public health activities in disaster settings at the national level, of which surveillance is understood to play a prominent role.^14^^,^ 15 ^,^ 16 While guidance particularly at the national level includes population assessment and surveillance, practices often do not conform to standardized reporting and assessment templates such as the Rapid Needs Assessment or the Utstein template for health disaster management.^12^^,^ 15

Despite the lack of conformity to a given reporting or surveillance template, a growing number of studies have been published in the field of health emergency management.^17^ We wished to explore how current practices of surveillance in the literature could be organized for use by local public health practitioners to ensure relevance and applicability to event type and scope. The goal of this study was to develop and test the reliability of a framework of current practices based on characteristics of disasters as well as surveillance methods. Determining which types of surveillance systems are best utilized at a given point in a natural disaster was viewed as a key step in enhancing current public health emergency management plans and evidence-informed decision-making. Specific objectives were to a) characterize relevant parameters of natural disaster events, including health outcomes and b) determine which modes of surveillance are best incorporated and useful within this classification system. Ultimately, this tool could become a surveillance resource for practitioners in local public health departments to access in the preparedness, response and recovery phases of a natural disaster.

## Methods

**Development of the Framework**

Based on a literature review of best practices in disaster classification, the framework was developed along four axes: the type of disaster, phase of the disaster cycle, extent of the damage, and anticipated health outcomes.

The type of natural disaster such as hurricane, tornado, ice storm, earthquake, flood, etc was categorized utilizing an internationally-recognized disaster category classification system.^3^ For the second axis, accepted definitions of the phases of disasters (impact phase, emergency phase and rehabilitation/recovery phase) were adopted.^18^ Although additional phases of non/inter-disaster and pre-disaster management are also described in the cycle, the focus was on the aforementioned phases for the current scope of work. In future, however, it is anticipated that emergency preparedness and mitigation practices would be informed by surveillance parameters and practices described in this study.

The extent of damage to health, infrastructure and the economy was classified using impact rating systems that are commonly utilized to assess risk associated with potential and real disasters. Within the frameworks examined from published literature, similar themes were found pertaining to human impact (fatalities or injuries); physical impact or property damage; and economic/social impact (including critical infrastructure and resources). Categories were developed based on severity of impact and combined into an overall impact rating which was scored based on review of previously used frameworks and discussion with content experts.^19^^,^ 20 ^,^ 21 ^,^ 22 This framework can be found in Appendix 1. The fourth and final classification axis was the main surveillance area being studied. For this section, a literature review was conducted to assess the most common types of surveillance associated with the types and phases of the disaster.

The literature search utilized two databases (Medline and EMBASE) and was limited to original studies on humans from 1980, the approximate time the field of disaster epidemiology began to develop, and extending to 2010.^18^ Natural disaster threats relevant to Ontario were included; those not relevant (for example, volcanoes, avalanches or tsunamis) were excluded.^23^ Non-English language articles were examined by abstract. Reviews were included in the literature search for purposes of checking reference lists but framework assessment was not completed for review articles. Articles were retrieved from the areas of public health/disaster planning; surveillance/disaster epidemiology; and natural disaster using relevant medical subject headings (MeSH); determination of which was assisted by information specialists. The MeSH headings were combined using Boolean logic. These search terms are provided in Appendix 2.

Search results were initially screened by title for relevance to public health surveillance by one reviewer; relevance at this stage was ascertained by general relevance to the study, namely surveillance and natural disasters. The process erred on the side of over-inclusion at this stage and subsequently, search results were examined by abstract for relevance to the study objective by two independent reviewers by more stringent criteria related to the specific study objective. Level of agreement between the two reviewers was determined using the kappa statistic for extraction by abstract.

Utilizing approximately 25% of articles as a pilot, the classification framework was finalized. Twenty-five percent of the most recent articles found in the literature search were chosen given the observation that the field of disaster epidemiology has expanded over time. Each article was examined for its subject area which was used to organize the studies by type. It was anticipated that the majority of articles would fall under several key outcomes for these areas, for example, studies of injuries or rapid health assessments. One author examined the articles to establish key areas (a), which was then reviewed by a second author (b) and finalized by consensus discussion on the 25% pilot articles.

**Testing the Reliability of the Framework**

The next phase of the study assessed the reliability of the framework by applying it to events described in the literature. This process was initiated by conducting a review of the literature. Henceforth, the term “reliability” will be used in reference to this phase of the study.

Using the same literature search described above, the methods for entering studies into the finalized four axis framework were as follows. For each natural disaster, the event was classified by disaster type (e.g. earthquake). Then, individual articles were examined for the phase of disaster to which the study pertained, and for each natural disaster event for which an article had been retrieved, the event was categorized by impact. Information on impact parameters for specific historical natural disasters was obtained from The International Disaster Database, a publicly accessible database maintained by the Centre for Research on the Epidemiology of Disasters.^24 ^ If impact data were not found in the database on all parameters, government websites were accessed for further information [e.g. Federal Emergency Management Agency, United States of America (USA)]. Finally, the key area its research pursued was classified as the last axis in the framework.

Of methodological note, articles that describe surveillance for populations that migrated from their original residence location (e.g. surveillance in evacuation centres) were classified according to the origin disaster type and impact. Additionally, articles were only classified once; thus, the best subject area or exposure was chosen. For example, an article describing injuries and illnesses after a disaster would be classified under basic descriptive study, rather than under injuries alone.

One reviewer (a) examined all articles retrieved by literature search and entered the articles into a template. The final template of studies and references was organized into a multi-layer Microsoft Excel™ Version 97-2003 (Microsoft™: Redmond, Washington) spreadsheet. Approximately 25% of articles were assessed by a second reviewer (b) for both impact score and article classification into the template. Percent agreement was determined for this 25% and differences were resolved by consensus discussion.

## Results

**Development of the Framework**

Based on a review of the classification systems and existing risk analysis tools, a sample framework was developed based on disaster type and impact, as demonstrated in Table 1.

**Table 1: Sample Framework:** 

REHABILITATION PHASE	Impact Severity of Event (Earthquake)			
EMERGENCY PHASE	Impact Severity of Event (Earthquake)			
IMPACT PHASE	Impact Severity of Event (Earthquake)			
Surveillance Area	1 Marginal	2 Serious	3 Critical	4 Catastrophic
area x				
area y				
area z				

After assessing the approximately 25% pilot articles, the finalized subject areas for surveillance were determined. These relevant areas are listed in Table 2. Studies were entered into the framework during the reliability phase based on disaster characteristics of type and impact factor, and study characteristics of phase of disaster and key area. The finalized template is displayed in Table 3.

**Table 2: Key Surveillance Areas Identified in the Literature:** 

Theme	Examples
Basic descriptive studies	Mortality or morbidity
Injuries	Emergency department visits for injuries
Environmental exposure	Carbon monoxide poisoning, hyperthermia
Infectious disease	Enteric outbreaks, other viral outbreaks
Chronic disease	Prescription medication needs
Rapid needs assessment	Cluster analysis technique
Vulnerable populations	Elderly
Other (including novel methods)	Use of cellular phones to transmit surveillance information

**Table 3: Finalized Classification Framework:** 

REHABILITATION PHASE	Impact Severity of Event (Earthquake)			
EMERGENCY PHASE	Impact Severity of Event (Earthquake)			
IMPACT PHASE	Impact Severity of Event (Earthquake)			
Surveillance Area	1 Marginal	2 Serious	3 Critical	4 Catastrophic
Basic descriptive studies				
Injuries				
Environmental exposure				
Infectious disease				
Chronic disease				
Rapid needs assessment				
Vulnerable populations				
Other (including novel methods)				

**Testing the Reliability of the Framework**

Medline search strategy resulted in 2384 citations with an additional 689 non-duplicate EMBASE citations, for a total of 3073. After initial assessment for relevance by title only, 394 citations remained. The independent review by two authors of these 394 citations by abstract resulted in good agreement with a kappa statistic of 0.80. Three hundred and fourteen (314) articles were agreed upon unanimously with the remainder resolved by consensus discussion. It was determined that saturation in content was achieved with inclusion of English articles; non-English articles that were reviewed by abstract were not felt to be substantially different from the group. Thus, further translation and analysis of non-English articles was not pursued. Final agreement between reviewers based on abstract resulted in 85 articles.

Twenty articles (23.5% of final total) were examined in order to assess the applicability of the framework.^25^^,^ 26 ^,^ 27 ^,^ 28 ^,^ 29 ^,^ 30 ^,^ 31 ^,^ 32 ^,^ 33 ^,^ 34 ^,^ 35 ^,^ 36 ^,^ 37 ^,^ 38 ^,^ 39 ^,^ 40 ^,^ 41 ^,^ 42 ^,^ 43 ^,^ 44 ^,^ 45 The percent agreement between reviewers in utilizing the framework was 74%.

The remainder (65) articles were input into the framework by one reviewer. A flowchart depicting the selection process for articles is provided in Appendix 3. In general, a description of the entirety of studies utilized for the reliability phase reveals that the majority of studies (58%) were on the subject of hurricanes, followed by earthquakes (16%). The majority of studies by impact fell into the category of either catastrophic or critical (81%) and 67% of studies were conducted during the emergency phase of the disaster. Thirteen percent of studies were rapid health assessments utilizing the Centers for Disease Control (CDC)/WHO-endorsed methodology of cluster analysis; 53% of studies were general (non-cluster analysis) descriptions of health needs and outcomes, including morbidity and mortality. The remaining articles were generally descriptive studies in various subject areas. Areas were noted to evolve over time to include relevant novel approaches, such as internet-based surveillance and use of cellular phones in surveillance.^25^^,^ 27 An example of the final completed framework using earthquakes in the emergency phase is demonstrated in Table 4.^25^^,^ 46 ^,^ 47 ^,^ 48 ^,^ 49 ^,^ 50 ^,^ 51 ^,^ 52 ^,^ 53

**Table 4:** 

EMERGENCY PHASE	Impact Severity of Event (Earthquake)			
Surveillance Area	1 Marginal	2 Serious	3 Critical	4 Catastrophic
**Basic descriptive studies**			(Carr, Leahy, London, Sidhu, & Vogt, 1996^47^)	(Akbari, Farshad, & Asadi-Lari, 2004^46^ ; De Bruycker et al., 1985^50^ ; Helminen, Saarela, & Salmela, 2006^51^ ; Pawar, Shelke, & Kakrani, 2005^52^ )
**Injuries**				(Roces, White, Dayrit, & Durkin, 1992^53^ )
**Environmental exposure**				
**Infectious disease**				
**Chronic disease**				
**Rapid needs assessment**				(Chen, Chen, Malilay, & Twu, 2003^48^ ; Daley, Karpati, & Sheik, 2001^49^ )
**Vulnerable populations**				
**Other (including novel methods)**				(Yang, Yang, Luo, & Gong, 2009^54^ )

## Discussion

Natural disasters with impact on human health and well-being can leave a jurisdiction in a varying state of disarray with significant pressure to respond to real, perceived or potential health threats. While it is important to respond quickly, it has also been shown that public health emergency response based on an assessment of health needs is more effective.^18^ Given that the field of disaster epidemiology is evolving and may not be familiar to all local public health practitioners, a resource of disaster surveillance and epidemiology would be useful. Using the four axes described; namely, type of disaster, phase of disaster, impact and major outcomes, this study created a framework where relevant surveillance systems can be classified. This study also demonstrated reliable classification of studies within this framework among two authors.

The potential use of this framework is three-fold. One is that it creates a resource table of surveillance systems that can direct people to the existing literature in a rapid and effective manner. This first is practical: if a jurisdiction is planning for or facing a major disaster with a potential human health impact and a surveillance system is required, the table directly links the responsible persons to the relevant literature. While the database currently represents the literature base reviewed as of 2010, and for disasters relevant to the authors’ jurisdiction, the development of a live, web-based, open-source database would allow an ongoing classification of the up-to-date literature, with contributions from global experts. Different surveillance approaches for health outcomes could be quickly reviewed and considered for implementation.

Secondly, over time, this classification may lead to a better understanding of the best practices in conducting surveillance during an emergency response. If a particular method is routinely shown to perform well with respect to important characteristics, it might become standard operating practice, and be relevant to the type and scope of a local event for which it has been shown to be effective. Finally, the framework may be used to identify gaps where research on the optimal surveillance methodology needs to be done.

The disaster type, phases of the disaster cycle, and severity of a disaster’s impact formed the basis of this framework. In reality, each disaster may not fit neatly into every category. For example, during the reliability component of the study it was found that some studies cannot be classified clearly within the “emergency phase” or “rehabilitation/recovery phase” but fall somewhere in a transition zone between. This could generally be seen to occur in an actual event, as a disaster transitions from one phase to another. It was decided which phase was most relevant for purposes of study classification, based on when the data was collected and whether the data would have been available in a particular phase. Additionally, certain areas of study in the literature evolved. Creating the category called “novel methods” utilized in surveillance could be a meaningful category for public health practitioners. However, it is recognized that what is novel today may not be novel in a few years time. For this framework, novel methodologies were defined by a new development in a surveillance system or the application of an existing surveillance system in a new way.

There are several limitations of this review and synthesis. Firstly, the focus was on natural disasters only, rather than assuming an “all-hazards” approach. Much of current emergency management practice emphasizes the need for an “all-hazards” approach, which the authors support; however, it was chosen to focus on the preliminary development of a framework and literature review on natural disasters in order to pilot the strategy and achieve an initially feasible goal. It is hoped that this framework could be applied to additional hazards in the future. Secondly, the literature review that was used for validation of the database is primarily focused on the published and indexed literature until 2010. It is likely that more information has been gathered on surveillance of health needs during and after natural disasters, with not all having been published or may be found within the “grey literature”. With focusing on the published literature, a sample of epidemiological research on this topic was sought in order to inform public health emergency preparedness in Ontario. Further validation could be done to increase the scope of hazards and also number of studies. This would increase the generalizability of this methodology.

A third limitation is that the majority of published studies described severe or catastrophic disasters, particularly hurricanes, were published in the last decade, and were conducted in the USA. While this is not surprising, it means that there was limited opportunity to apply the framework to less severe disasters and those in non-USA settings. On the other hand, the framework will likely be most useful in large scale disasters. Fourth, the impact rating, while developed from previously published classification scales, is subject to bias given the qualitative nature of the assessment components. However we tested the reliability of the scales when testing the framework itself and found the percent agreement to be high when performed independently by two authors. Finally, the framework is still in development and is based on a limited number of studies. Utility and relevance of the developed resource will need to be tested and evaluated for generalizability by the stakeholders for whom it was designed: namely, local public health. Furthermore, in order to be relevant to the evolving nature of disaster epidemiology as natural disasters continue to occur around the globe, ongoing review of the literature needs to be conducted and the database should be updated in a live modality while accessible to local public health. Utilizing the internet to establish an internet database would allow this functionality.

While the authors recognize the limitations of this study, it is viewed as an important building block for enhancing surveillance capacity for emergency settings at the local level. Future work could include validating the framework at the local level in either real-world natural disaster settings or through simulation methodology employing natural disaster scenarios.

## Conclusions

A framework was developed that can allow local public health practitioners to access the appropriate and previously utilized surveillance methods for a natural disaster in a rapid fashion. The largely descriptive analyses were organized into a database by disaster characteristics and type of study in order to classify and create an analytic framework. It is felt that in a rapidly evolving disaster, an accessible and structured database of surveillance knowledge can inform best practices to target the actual health needs of the affected population. While the focus was on literature conducted at the time of or in the aftermath of a disaster, the database is conceptually viewed as a resource to be accessed during emergency preparedness and planning to facilitate an evidence-informed epidemiology component to emergency plans. It is important, however, that larger organizations such as national, provincial/state and academic institutions continue to support public health capacity in conducting disaster research in analytic methodologies. Further research is needed to evaluate the utility of the developed surveillance framework in the local public health setting.

## Competing Interest

The authors report no competing interests exist.

## Appendix 1

### Framework to Classify Disasters Based on Overall Impact

References:^19^ ^20^ ^21^ ^22^ 

**Human Impact (Fatalities):**

Category	Assessment	Description
1	0-4	Very low
2	5-10	Low
3	11-50	High
4	>50	Very high

**Physical Impact (Property Damage):**

Category	Assessment	Description
1	Minimal damage	Very low
2	Localized damage	Low
3	Severe damage	High
4	Widespread damage	Very high

**Critical Infrastructure & Resources and Economic & Social Impact:**

Category	Assessment	Description
1	Temporary interruption/impact	Very low
2	Temporary and widespread	Low
3	Extended and widespread	High
4	Permanent impact	Very high

**OVERALL IMPACT RATING:**

**Category**	**Score**	**Description**
1	3-4	Marginal
2	5-7	Serious
3	8-10	Critical
4	11-12	Catastrophic

## Appendix 2

### Final Search Terms Utilized for Medline and Embase Database Searching

Download PDF

## Appendix 3

### **Flowchart Depicting Selection of Articles for Developing and Testing Framework**

Download PDF
